# Patients’ acceptance of a shared cancer follow-up model of care between general practitioners and radiation oncologists: A population-based survey using the theoretical Framework of Acceptability

**DOI:** 10.1186/s12875-023-02032-6

**Published:** 2023-03-28

**Authors:** Tiffany Sandell, Heike Schütze, Andrew Miller, Rowena Ivers

**Affiliations:** 1 Graduate School of Medicine, Faculty of Science, Medicine and Health, University of Wollongong, Wollongong, NSW Australia; 2 Cancer Services, Illawarra Shoalhaven Local Health District, Wollongong, NSW Australia; 3 Centre for Primary Health Care and Equity, Faculty of Medicine, UNSW, Sydney, NSW Australia

**Keywords:** Shared care, Primary care, Radiation oncology, Theoretical Framework of Acceptability, General practitioner, cancer follow-up care

## Abstract

**Introduction:**

International and national guidelines highlight the need for general practitioner involvement during and after active cancer treatment and throughout long-term follow-up care. This paper aimed to evaluate patients’ acceptance of radiation oncology shared follow-up care using the Theoretical Framework of Acceptability (TFA).

**Methods:**

This cross-sectional study was conducted at two cancer care centres in the Illawarra Shoalhaven region of Australia. A sample of patients scheduled for a radiation oncology follow-up consultation in 2021 were sent a 32-point self-complete paper-based survey. Data were analysed using descriptive, parametric and non-parametric statistical analysis. This paper followed the Checklist for Reporting of Survey Studies (CROSS).

**Results:**

Of the 414 surveys returned (45% response rate), the acceptance for radiation oncology shared cancer follow-up care was high (80%). Patients treated with only radiotherapy were 1.7 times more likely to accept shared follow-up care than those treated with multiple modalities. Patients who preferred follow-up care for fewer than three years were 7.5 times more likely to accept shared care than those who preferred follow-up care for five years. Patients who travelled more than 20 minutes to their radiation oncologist or to the rural cancer centre were slightly more likely to accept shared care than those who travelled less than twenty minutes to the regional cancer centre. A high understanding of shared care (Intervention Coherence) and a positive feeling towards shared care (Affective Attitude) were significant predictive factors in accepting shared radiation oncology follow-up care.

**Conclusion:**

Health services need to ensure patient preferences are considered to provide patient-centred cancer follow-up care. Shared cancer follow-up care implementation should start with patients who prefer a shorter follow-up period and understand the benefits of shared care. However, patients’ involvement needs to be considered alongside other clinical risk profiles and organisational factors. Future qualitative research using the TFA constructs is warranted to inform clinical practice change.

**Supplementary Information:**

The online version contains supplementary material available at 10.1186/s12875-023-02032-6.

## Introduction

Once cancer patients complete active treatment, they require long-term follow-up to monitor for treatment-related side effects, recurrence, and psychosocial support [1–3]. The usual model of care is the hospital-based oncologist-led model [4, 5]. There is usually little formal involvement with the patient’s general practitioner in this model of care [6, 7]. While the oncologist-led model suits many patients, it may not always meet patients’ physical and psychosocial needs [8, 9]. For some patients, a shared care model might be more appropriate, tailored to their tumours, treatments, locality (metropolitan, regional or rural), access to specialists, and specific physical and emotional needs and preferences [10].

Shared care differs from the partial or whole transfer of care, where aspects of care are wholly transferred from the oncologist to another provider, such as a general practitioner. A shared cancer follow-up model of care harnesses the expertise of health professionals [11] and involves the explicit sharing of information and coordination of follow-up care [12]. Shared care is widely used in antenatal care and for managing patients with asthma, diabetes and ischaemic heart disease [13–16].

There is a growing body of evidence supporting the benefits of shared cancer follow-up models of care [17–21]. Randomised controlled trials have shown no difference in cancer recurrence rates or quality of life when a general practitioner is involved in follow-up care [22–25]. A shared cancer follow-up model of care offers several advantages to patients, health providers and health services. Patients generally find general practitioner appointments are more accessible than specialist appointments [26, 27]; there are fewer duplication of tests and clinical questions; reduced travel time; and more accessible parking [28, 29]. Benefits for general practitioners include increased knowledge and awareness of their patient’s overall health [30], addressing their patient’s unmet psychosocial needs [31], and improving relationships with specialists [32]. A shared care model benefits oncologists by allowing more time for newly diagnosed patients, patients on active treatment, advanced-stage and complex patients [33], and involvement in research and development activities. Additionally, the cost of shared cancer follow-up care to the health system is less than standard oncology follow-up care [34, 35].

A shared cancer follow-up model of care may vary for each medical discipline (medical oncology, radiation oncology, haematology, surgical) and tumour type (breast, prostate, head and neck, abdomen, lung, etcetera). For example, in one model for shared care for colorectal patients, the general practitioner and oncologist alternate the appointments quarterly [36]. In another model specific to radiation oncology shared follow-up care for breast, prostate and colorectal cancer patients, the radiation oncologist consultations cease entirely after three years after treatment, and patients see their general practitioner [37]. In that model the general practitioner follows a prescribed clinical follow-up assessment, and the radiation oncologist oversees and reviews the consultation results; this model is reliant on health technology and the two-way transfer of clinical information in real-time [37].

A core principle of shared cancer follow-up care is the acceptability of all parties: the oncologist, the general practitioner and the patient [38]. General practitioners are willing to accept a greater role in cancer follow-up care if there is improved information sharing and they are provided with clear clinical follow-up guidelines or protocols [39–41]. However, increased workload concerns remain [42–44]. Oncologists are more likely to prefer an oncologist-led model instead of a shared care or general practitioner-led model, as they feel they have the specialised knowledge for follow-up care [45]. However, oncologists are receptive to general practitioners taking a greater role in the more standardised aspects of follow-up care for low-risk patients [6], such as managing long-term and late side effects, blood tests and physical examinations. Several qualitative studies have found that patients appreciate their general practitioners taking a greater role in their long-term care [9, 29, 41, 46, 47].

Despite increasing evidence of the effectiveness of shared cancer follow-up care, data on implementation is limited. Treatment types (chemotherapy, immunotherapy, radiotherapy, surgery, etcetera) cause different short-term and long-term side effects [48], and differences in acceptance based on treatment type may help inform implementation. However, there is limited quantitative research on patients’ acceptance of a shared cancer follow-up model of care specific to radiation oncology patients, to allow generalisability to larger samples. This study aimed to evaluate patients’ acceptance of a shared cancer follow-up model of care between their general practitioner and radiation oncologist using the Theoretical Framework of Acceptability, in the Illawarra Shoalhaven Local Health District.

## Methods

### Study design, setting and participants

The Checklist Reporting of Survey Studies (CROSS) guided this study (Supplementary file 1). This cross-sectional study used a purpose-developed survey and was set in one regional, Illawarra Cancer Care Centre, and one rural, Shoalhaven Cancer Care Centre, Australia. This region provides health services for around 400,000 people, including radiotherapy-related services for over 6,000 distinct people for treatment and consultations annually. The study population was patients on a radiation oncology follow-up regime at one of these cancer centres in 2021.

In Australia, oncologists are guided by the American Society of Clinical Oncology, the National Comprehensive Cancer Network, and the National Institute for Clinical Excellence guidelines for follow-up care. Follow-up care is between five to 10 years, however, the actual frequency depends on the individual patient’s health, stage,treatment, and an oncologist’s individual approach.

### Data sampling and data collection

In 2021, there were 6,036 distinct patients scheduled for radiation oncology follow-up appointments in the study sites. We calculated that three-hundred and sixty-two completed surveys were required to obtain a 95% confidence interval, with a 5% margin of error. We anticipated a 40% response rate. This was assumed because paper-based surveys obtain response rates of 46% compared to online surveys of 36% [49], and we were using a mixture of the two. Therefore, we sent 950 paper-based surveys and patients could elect to return the paper survey in the prepaid envelope provided or complete the survey online using the provided Quick Response Code or weblink. A proportional stratified random sampling approach was employed based on years since treatment. Years since treatment strata were divided into < 1 year, 1–2 years, 3–4 years, 5 years, 6–10 years and > 10 years. The sample from each stratum was randomly selected using a Microsoft Excel formula to generate the participant list.

### Survey

A 32-point survey was developed by the authors and comprised four sections: demographics, health and cancer-related information, access to healthcare, and acceptance of shared care. The options in the demographics, health and cancer-related information, and access to healthcare sections were adapted based on previous survey designs [50–52], and are described below.

Demographics: These included age, sex, postcode, country of birth, primary language spoken, ethnicity, relationship status, level of education, housing situation, employment status, and income.

Health and cancer-related information: The cancers with the highest incidence [48] (breast, prostate, lung, colorectal, pelvis and head and neck) were included, as well as an ‘Other’ option. Additional information included: the staging at diagnosis, the primary hospital where radiation oncology treatment was received, other treatments received, and years post active treatment. A five-point scale ranging from ‘Excellent’ to ‘Poor’ based on World Health Organisation recommendations  [53] was used to measure self-reported health status.

Access to healthcare: Questions included whether the patient had a regular general practitioner, how often they visited their different doctors, the time required to travel to their doctors and the primary mode of transport, and how often they would like a consultation for their radiation oncology follow-up care, and how many years they would prefer follow-up care.

Acceptability of shared cancer follow-up care: Acceptance of shared care was based on the Theoretical Framework of Acceptability (TFA) [54]. The TFA is a multi-construct theoretical framework designed explicitly to assess the acceptability of healthcare interventions from the perspectives of the people who receive the intervention and those who deliver it [54]. The TFA can be applied quantitatively or qualitatively and used prior to a health intervention to form judgments about whether the participants expect the intervention to be acceptable or unacceptable. Assessment of anticipated acceptability prior to participation can highlight which aspects of the intervention could be modified to increase acceptability and thus, participation [54]. The seven constructs of the framework used to determine overall acceptability are Affective Attitude, Burden, Ethicality, Intervention Coherence, Opportunity Costs, Perceived Effectiveness and Self-efficacy. Questions were developed based on these constructs and measured using a five-point Likert scale from ‘Strongly disagree’ to ‘Strongly agree’ (see Table 1 for example).

**Table 1:** Theoretical Framework of Acceptability constructs and example statements used in the questionnaire

TFA Constructs	Definition	TFA questions on 5-point Likert scale (Strongly disagree to Strongly agree)
**Affective Attitude**	Anticipated Affective Attitude: how an individual feels about the intervention, prior to taking part.	I would be **satisfied** for my radiation oncology follow-up care to be shared with my general practitioner, so long as the radiation oncologist is still involved.
**Burden**	Anticipated burden: the perceived amount of effort that is required to participate in the intervention.	It is **easier** to get to my general practitioner than the hospital. (Transport, time, parking, accessibility)
**Ethicality**	The extent to which the intervention has good fit with an individual’s value system.	I would **value** my radiation oncologist and general practitioner working together to share my follow-up care.
**Intervention Coherence**	The extent to which the participant understands the intervention and how it works.	I **understand** that shared radiotherapy follow-up care will benefit me, my doctors and the health system.
**Opportunity Costs**	Anticipated opportunity cost: the extent to which benefits, profits, or values must be given up to engage in the intervention.	In order to have shared follow-up care, I would **need to give up** some of my time or my values on shared-care.
**Perceived Effectiveness**	Anticipated effectiveness: the extent to which the intervention is perceived to be likely to achieve its purpose.	I **believe** that shared radiotherapy follow-up care will benefit me, my doctors and the health system.
**Self-efficacy**	The participant’s confidence that they can perform the behaviour(s) required to participate in the intervention.	If I had the choice: a. I would choose to have my follow-up care with **only** my radiation oncologist. b. I would choose to have **shared** follow-up care with my general practitioner so long as my radiation oncologist is involved. c. I have **confidence** in my choice above.

The survey was refined with feedback from four general practitioners and two radiation oncologists. The survey included as few questions as possible to reduce the burden on patients and improve the response rate [55]. Readability was scored at Year Nine level, which is slightly higher than the Australian desired standard of Years Six to Eight [56]. The survey was piloted on ten follow-up patients and ten cancer centre staff for content validity. The average completion time was 4.5 minutes. The final version was available in printed form and online using Qualtrics XM.

### Reliability and validity

Internal consistency reliability estimates how much total test scores would vary if slightly different items were used [57]. The reliability across the seven constructs was assessed by computing Cronbach’s α, with the minimum acceptable value of α = 0.70. The coefficients for the constructs totalled α = 0.78, indicating an acceptable level of internal consistency  [58, 59]. Table 2 shows that the reliability would slightly improve if the Opportunity Costs construct were removed; however, the research team deemed the improvement small and did not delete it. The construct validity of the TFA constructs (that is, how accurately the constructs measure what they say they do)  [60] was calculated with Pearson’s correlation coefficient of the patient’s responses to an item with their total scores. A validity coefficient above 0.35 is strongly valid [61], and all constructs were positively associated.

**Table 2:** Reliability and validity analysis of acceptability constructs

	Corrected item total correlation	Cronbach’s Alpha if item deleted	Pearson correlation	Sig (2-tailed)
Affective Attitude	0.603	0.727	0.684	< 0.001
Burden	0.182	0.819	0.373	< 0.001
Ethicality	0.756	0.699	0.764	< 0.001
Intervention Coherence	0.753	0.704	0.787	< 0.001
Perceived Effectiveness	0.772	0.695	0.796	< 0.001
Opportunity Costs	0.281	0.803	0.523	< 0.001
Self-Efficacy	0.373	0.771	0.413	< 0.001

### Statistical analysis

Data were analysed using the statistical software package SPSS version 29 [62]. Frequencies and percentages were calculated for categorical variables and reviewed for normality. Two forms of acceptance scores were generated. For the first, an average score across all TFA constructs, with opportunity scores reversed to align from a negative to a positive scale. A patient’s summated score was divided by the number of constructs constituting the scale, thereby creating a mean that falls within the range of the values for the response continuum options. All items comprising the construct’s scale were assumed to have equal weight when calculating a summated score. The second form of acceptance score was achieved by dichotomising the data into ‘Accept’ and ‘Not Accept’. The Accept score was generated from the ‘Strongly agree’ and ‘Agree’ response categories, and the Not accept score was generated from ‘Neither agree/disagree’, ‘Disagree’ and ‘Strongly disagree’. The dichotomised data were used to understand whether acceptance could be predicted based on any of the TFA categorical constructs (logistic regression).

Parametric tests included multinomial and ordinal logistic regression. If requirements for parametric test procedures were not met, non-parametric tests were used: Chi-Square, Kruskal Wallace Test and posthoc analysis. All tests were 2-sided; statistical significance was defined as *p* ≤ 0.05. Weighted adjustments were used to compensate for missing data.

### Ethical consideration

Ethical approval for this study was obtained from the Joint University of Wollongong and Illawarra Shoalhaven Local Health District Human Research Ethics Committee. Patients were provided with a participant information sheet about the study’s aim and procedures and informed that consent was tacit upon completing the survey and that responses would be anonymised. Patients did not receive payment or an honorarium.

## Results

Of the 950 surveys sent, 414 were returned (response rate of 45%); 371 had no missing data. Twenty-eight surveys were returned to sender (26 were no longer at that address, and two stated that the patient was deceased). Most (383 of 414) surveys were returned via post (92%). Patient demographics, health characteristics and healthcare access are presented in Table 3. Age and sex did not significantly influence a patient’s preference regarding their choice of follow-up care, and there were no significant socio-demographic variables on the acceptance for shared care.

**Table 3:** Demographics of radiation oncology respondents

	N (%)		N, (%)
**Sex**		**Cancer**	
Male	171, (41.3)	Breast	193, (46.6)
Female	243, (58.7)	Colorectal	9, (2.2)
		Head/Neck	54, (13)
		Lung	25, (6)
		Prostate	91, (22)
		Pelvis	9, (2.2)
		Other	33, (8)
**Age**		**Stage at diagnosis**	
< 40	5, (1.2)	I	91, (22)
41–50	38, (9.5)	II	76, (18.4)
51–60	13, 2(32.9)	III	57, (13.8)
61–70	201, (50.1)	IV	25, (6)
> 71	25, (6.2)	Not known	165, (41.1)
**Education**		**Treatment**	
Year 10	126, (31.2)	Only radiotherapy	141, (34.1)
Year 12	42, (10.4)	Radiotherapy and other	273, (65.9)
Certificate	117, (29)		
Undergraduate	43, (10.6)		
Postgraduate	52, (12.9)		
Prefer not to say	24, (5.9)		
**Relationship**		**Years since treatment**	
Married	278, (67.5)	Less than 1 year	122, (29.8)
Single	25, (6.1)	1–2 years	82, (20)
De-facto	13, (3.2)	2–3 years	59, (14.4)
Divorced	37, (9.0	3–4 years	51, (12.5)
Widow	53, (12.9)	4–5 years	45, (11)
Prefer not to say	6, (1.5)	5–10 years	43, (10.5)
		> 10 years	7, (1.7)
**Housing**		**Health**	
Rent	48, (11.7)	Excellent	46, (11.3)
Own	341, (82.8)	Very good	131, (32.2)
Other	14, (3.4)	Good	149, (36.6)
Prefer not to say	9, (2.1)	Fair	68, (16.7)
		Poor	13, (3.2)
**Employment**		**Main hospital treated at**	
Casual	15, (3.7)	Illawarra Cancer Care Centre - Regional	262, (64)
Part-time	40, (9.8)	Shoalhaven Cancer Care Centre - Rural	147, (36)
Full-time	32, (7.8)		
Unable to work	18, (4.4)		
Retired	295, (72.1)		
Prefer not to say	9, (2.2)		
**Income**		**Travel time to General practitioner (one way)**	
<$15,000	58, (15.2)		
$15,000–29,999	97, (25.5)	0–20 minutes	361, (90.5)
$30,000–49,000	63, (16.5)	21–40 minutes	27, (6.7)
$50,000–74,999	46, (12.1)	> 40 minutes	11, (2.8)
$75,000-100,000	23, (6)		
> 100,000	10, (2.6)		
Prefer not to say	84, (22)		
**Country of birth**		**Travel time to Radiation oncologist (one-way)**	
Australia/New Zealand	324, (78.3)		
United Kingdom	46, (11.1)	0–20 minutes	156, (40.1)
Europe	31 (7.5)	21–40 minutes	159, (40.9)
Africa	5 (1.2)	41–60 minutes	51, (13.1)
Asia	5 (1.2)	> 1 hour	12, (3.1)
Canada	3 (0.7)	> 2 hours	11, (2.8)
**Primary language**		**Preferred radiation oncology frequency**	
English	404, (97.6)	Every second month	11, (2.8)
Other	10, (2.4)	Every three months	48, (12.1)
		Every six months	108, (27.3)
		Once a year	167, (42.2)
		No more visits wanted	62, (15.7)
**Identifies as Aboriginal and/or Torres Strait Islander**		**Preferred year for follow-up**	
		No follow-up wanted	38, (9.6)
		For 1 year	67, (17)
No	398 (98)	For 3 years	32, (8.1)
Yes	9 (2)	For 5 years	174, (44.1)
		For 10 years	47, (11.9)
		For lifetime	37, (9.4)

One-third of the patients (n = 141, 34%) were treated with only radiotherapy, and two-thirds (n = 273, 66%) were treated with radiotherapy and chemotherapy and/or surgery. More patients reported their health as either ‘Excellent’ or ‘Very good’ (43.5%), followed by ‘Good’ (36.6%) and ‘Fair’ or ‘Poor health’ (19.9%). Almost all patients had a regular general practitioner (98%); 90% lived within a 20-minute drive of their general practitioner, and 40% lived within a 20-minute drive of their radiation oncologist.

Table 4 shows a high acceptance of radiation oncology shared care across the different tumour types. However, no statistically significant results were found with patient acceptance of shared care between the tumour group, cancer staging, or years since treatment.

**Table 4:** Acceptance for radiation oncology shared follow-up care

	Accept Shared Care N (%)	Do not accept shared care N (%)	N
**Total average acceptance**	**325, (80%)**	**79, (20)**	**405**
Breast	149, (79)	39, (21)	188
Colorectal	7, (78)	2, (22)	9
Head/Neck	44, (83)	9, (17)	53
Lung	20, (83)	4, (17)	24
Prostate	74, (82)	16, (18)	90
Pelvis	9, (100)	0, (0)	9
Other cancer	23, (72)	9, (28)	32

### Theoretical Framework of Acceptability Constructs

Table 5 shows patients’ acceptance for shared follow-up care across each construct in the Theoretical Framework of Acceptability. For Affective Attitude 85% agreed that they would be satisfied for their follow-up care to be shared with their general practitioner as long as the radiation oncologist was still involved. Ethicality: 88% agreed a shared cancer follow-up model fits with their values. Intervention Coherence: 88% agreed that they understood the benefits of shared cancer follow-up care for themselves, their doctors and the health care system. Perceived Effectiveness: 87% agreed that shared care was likely to achieve its purpose. Self-Efficacy: 75% elected to have shared follow-up care; sub-analysis showed 97% had confidence in their choice (*p* = < 0.001).

**Table 5:** Acceptance for radiation oncology shared follow-up care according to the Theoretical Framework of Acceptability constructs

TFA Constructs	Agree	Disagree
Affective attitude	85%	15%
Burden	30%	70%
Ethicality	88%	12%
Intervention Coherence	88%	12%
Perceived Effectiveness	87%	13%
Opportunity Costs	33%	77%
Choose Shared Care	75%	15%
Self-Efficacy	93%	7%

### Acceptance and preferences for follow-up care

Patients treated with only radiotherapy were associated with an increase odds of accepting shared care, odds ratio 1.707 (95% CI 1.051–2.773), Wald *χ*^2^(1) = 4.668, *p* < 0.031. Additionally, patients who self-reported ‘Very good’ health had a statistically significant higher acceptance of shared care than those who self-reported their health as ‘Good’ (*p* = 0.008). However, health status was not a strong predictor of accepting shared care (*χ*^2^(4), 7.951, p = 0.093).

The majority of patients preferred to have their radiation oncology follow-up for five years (44%). Patients who preferred follow-up care for one year were 2.9 times more likely to accept shared care (*p* = 0.025), than those who wanted follow-up care beyond five years; and those who preferred follow-up care for three years were 7.5 times more likely to accept shared care (*p* = 0.012) than those who preferred care beyond five years.

Patients treated at the regional hospital were 1.8 times more likely to want follow-up care to continue for over 10 years (*p* = 0.027), and five times more likely to want follow-up for life (*p* = < 0.001), compared to patients treated at the rural hospital. These results align with travel time. Patients who travelled less than 20 minutes one-way to their radiation oncologist had a slightly lower acceptance for shared care (mean rank = 186.08, *p* = 0.025) than those who travelled more than 20 minutes (mean rank = 207.15, *p* = 0.025). Although not significant, patients treated at the rural hospital had a slightly higher average acceptability score of shared care (3.94/5 compared to 3.86/5 from the regional hospital).

Logistic regression predicted patients’ acceptance of shared care (see Table 6). Patients with a high understanding of shared care (Intervention Coherence) were predicted to be seven times more likely to accept a shared cancer follow-up model of care; those with a high Affective Attitude were predicted to be three times more likely; those with a high Ethicality were two and half times more likely; and those with high Self-Efficacy were three times more likely. Other constructs were not significant in predicting acceptance of shared care.

**Table 6:** Odds ratio of Theoretical Framework of Acceptability and shared care acceptance

	df	Sig.	OR
Affective Attitude	1	< 0.001	3.231
Burden	1	0.306	1.131
Ethicality	1	0.031	2.497
Intervention Coherence	1	< 0.001	7.111
Opportunity Costs	1	0.162	0.824
Perceived Effectiveness	1	0.781	1.129
Self-Efficacy	1	0.007	3.467

## Discussion

This multi-centre cross-sectional study evaluated patients’ acceptance of a shared cancer follow-up model of care between their general practitioner and radiation oncologist using the Theoretical Framework of Acceptability (TFA). We found that 80% of patients accepted a radiation oncology shared follow-up model of care, and 75% would choose shared care compared to the oncologist-led model if given a choice. Patients treated only with radiotherapy were more likely to accept shared follow-up care, and patients who preferred follow-up care for fewer than three years were more likely to accept shared follow-up care. The TFA constructs of Intervention Coherence, Affective Attitude and Self Efficacy were significant predictors of acceptance for shared cancer follow-up care.

Previous qualitative research has found that patients are willing to accept shared cancer follow-up care if their oncologist remained remains involved and can oversee their care [6]. Although previous research does not distinguish between patients treated with only radiation therapy or other modalities, this study confirms that most radiation oncology patients would accept shared cancer follow-up care provided their radiation oncologist was still involved. However, the extent to how the oncologist was to remain involved was not explicitly addressed. It has been suggested that for the oncologist to remain involved and oversee the patient’s care, there is a need for improved two-communication and linkage of medical records between health professionals [6, 63].

Some patients require follow-up appointments with multiple specialists: radiation oncologist, medical oncologist, surgeon (for example, urologist, breast surgeon), and shared care has been highlighted as beneficial in reducing the number of appointments and duplication of assessments [6, 64]. However our results found that patients who only received radiotherapy treatment were more likely to accept shared follow-up care, and no significant difference with years since treatment was found. This is an interesting result, as patients treated with only one modality have fewer follow-up consultations than those treated with multiple modalities (who would be more likely to benefit from having fewer appointments). The higher acceptance for patients treated with only radiotherapy may be due to other unknown factors, such as long-term toxicity and treatment side effects and warrants further investigation.

To our knowledge, this is the first quantitative study to apply the TFA, which helped determine factors that may predict a patient’s acceptance of a radiation oncology shared follow-up model of care. Patients with good Intervention Coherence, Affective Attitude and Self-Efficacy were significantly more likely to accept a shared care model. Additionally, these constructs were also useful in predicting acceptance and could be useful for health services to undertake readiness assessments. This finding is also supported by the Social Cognitive Theory that goes beyond the individual behaviour (Health Belief Model and Theory of Reasoned Action/Planned Behaviour) and considers interactions with social and environmental influences. According to Bandura [65], if people lack awareness of how their lifestyle habits affect their health, they have little reason to change; conversely, knowledge creates the precondition for change [65]. Therefore, the knowledge and understanding (Intervention Coherence) regarding the benefits of shared cancer follow-up care is important to consider before transferring the care of patients to their general practitioner in a shared care model. This finding is also supported by a recent study that found women need to be provided with the evidence that shared follow-up care is effective, so they can form a thorough understanding (Intervention Coherence) of what shared is, who is responsible for what and to understand that shared care will not negatively impact their health outcomes [66]. The TFA allows researchers and health services to determine which constructs require further attention to increase acceptance before implementing health interventions.

Although there are several system barriers to implementing shared cancer follow-up care (such as the need for defined health professional roles [6], protocols, evidence-based guidelines [40, 46, 67], and communication tools [28]), acceptability to patients is fundamental. Our results support that shared cancer follow-up care needs to be individualised based on the patient’s cancer type, treatment type, current health, and personal preferences  [50]. The American Society of Clinical Oncology suggested that “models of risk are needed to stratify survivors into different levels of intensity and setting for follow-up care. Components needed in such a model include risk recurrence, the persistence of moderate to severe toxicity or therapy, risk of serious physical late effects and psychosocial status" [68 p.634]. Another form of stratification to select appropriate patients for a shared care model beyond the clinical paradigm is to evaluate the patient’s acceptability toward shared care.

In addition to the risk stratification for cancer patients, essential elements for shared care include improved communication between the general practitioner and oncologist [32, 69, 70]. It is equally important to provide patient-centred care, including engaging with patients and understanding their needs and preferences [71]. We show that patients with a strong understanding (Intervention coherence) of the benefits of shared care are seven times more likely to accept a shared care follow-up model.

### Study limitations

To our knowledge, this was the first study that used the Theoretical Framework of Acceptability quantitatively, and there is limited guidance on applying the framework in survey format. The study was specific to radiation oncology follow-up; some patients may have confused this with their medical oncology or surgical oncology follow-up. Although this study had a good response rate, there is no information about the 55% who declined to participate. It is possible that those who did not respond were less likely to accept shared cancer follow-up care, and response bias may be present. The authors were unable to conduct a non-report analysis. Additionally, there were few responses from colorectal cancer patients; this may be due to fewer colorectal cancer patients being treated with radiotherapy compared to breast and prostate patients. Finally, this study was conducted across a regional and rural population and may not be generalisable to the metropolitan population. However, based on our results, patients who travel less than 20 minutes to their oncologist were slightly less likely to accept shared care and may produce similar results in a metropolitan area where people live closer to cancer centres. The lead author is a critical realist researcher and acknowledges that many unobservable structures and events may influence the results.

## Conclusion

There is a need to normalise shared cancer follow-up care into practice. However, normalising shared cancer care requires a multifaceted approach and support from specialists, general practitioners and patients. Based on the findings of this study, informing patients about the concept and benefits of shared care is important to foster acceptance. Follow-up care should be based on individual clinical risk and patient preference for follow-up care. Further investigation is needed to establish how the oncologist is to remain involved and oversee care in a shared care model, and to qualitatively research the acceptance among radiation oncologists, general practitioners and patients using the TFA constructs to inform clinical practice change.

## Electronic supplementary material

Below is the link to the electronic supplementary material.

Supplementary Material 1

## Data Availability

The datasets generated during and/or analysed during the current study are available from the corresponding author upon reasonable request.
